# The clinical course of acute otitis media in high-risk Australian Aboriginal children: a longitudinal study

**DOI:** 10.1186/1471-2431-5-16

**Published:** 2005-06-14

**Authors:** Katherine B Gibney, Peter S Morris, Jonathan R Carapetis, Susan A Skull, Heidi C Smith-Vaughan, Elizabeth Stubbs, Amanda J Leach

**Affiliations:** 1 Department of Medicine, Royal Darwin Hospital, Australia; 2 Ear Health and Education Unit, Menzies School of Health Research, Darwin, Australia; 3 Institute of Advanced Studies, Charles Darwin University, Australia; 4 Northern Territory Clinical School, Darwin, Australia; 5 Department of Paediatrics, University of Melbourne and Royal Children's Hospital, Melbourne, Australia; 6 Murdoch Children's Research Institute, Melbourne, Australia

## Abstract

**Background:**

It is unclear why some children with acute otitis media (AOM) have poor outcomes. Our aim was to describe the clinical course of AOM and the associated bacterial nasopharyngeal colonisation in a high-risk population of Australian Aboriginal children.

**Methods:**

We examined Aboriginal children younger than eight years who had a clinical diagnosis of AOM. Pneumatic otoscopy and video-otoscopy of the tympanic membrane (TM) and tympanometry was done every weekday if possible. We followed children for either two weeks (AOM without perforation), or three weeks (AOM with perforation), or for longer periods if the infection persisted. Nasopharyngeal swabs were taken at study entry and then weekly.

**Results:**

We enrolled 31 children and conducted a total of 219 assessments. Most children had bulging of the TM or recent middle ear discharge at diagnosis. Persistent signs of suppurative OM (without ear pain) were present in most children 7 days (23/30, 77%), and 14 days (20/26, 77%) later. Episodes of AOM did not usually have a sudden onset or short duration. Six of the 14 children with fresh discharge in their ear canal had an intact or functionally intact TM. Perforation size generally remained very small (<2% of the TM). Healing followed by re-perforation was common. Ninety-three nasophyngeal swabs were taken. Most swabs cultured *Streptococcus pneumoniae *(82%), *Haemophilus influenzae *(71%), and *Moraxella catarrhalis *(95%); 63% of swabs cultured all three pathogens.

**Conclusion:**

In this high-risk population, AOM was generally painless and persistent. These infections were associated with persistent bacterial colonisation of the nasopharynx and any benefits of antibiotics were modest at best. Systematic follow up with careful examination and review of treatment are required and clinical resolution cannot be assumed.

## Background

Today, the majority of episodes of acute otitis media (AOM) in developed countries will resolve even if they are not treated with antibiotics. This conclusion is based on the findings of randomised placebo-controlled trials, longitudinal studies of initially withholding antibiotic treatment, and meta-analyses of randomised controlled trials. [1-3]. However, the outcomes in children from populations where there is an increased risk of complications remain uncertain.

We have described the onset of early and severe otitis media in Australian Aboriginal children[4,5]. This is associated with dense colonisation of the nasopharynx with respiratory bacteria. In previous studies in this population, AOM (defined as the presence of an effusion plus bulging of the tympanic membrane (TM) or recent perforation) was frequently asymptomatic. AOM was often present on otoscopic examination 4 weeks after onset, despite antibiotic treatment [6]. Children with frequent episodes of bulging of their TM were those most likely to develop new perforations. However, we could not determine whether AOM in high-risk populations initially responded to treatment and then recurred, or persisted despite treatment. The aim of this study was to describe the clinical course of AOM and the associated bacterial nasopharyngeal colonization in Aboriginal children from a remote community in the period immediately after diagnosis.

## Methods

### Setting

The local Human Research Ethics Committee and the community-controlled Tiwi Health Board approved the study. It took place in the year 2000 in a remote Aboriginal community situated 70 km north of Darwin (population 1300). The community has an average of 30 births per year and an infant mortality rate of 30 per 1000 live births. The standard of housing is poor and overcrowding is common [7].

### Participants

We enrolled Aboriginal children younger than eight years who lived in the community if: i) they had AOM; ii) they were resident in the community; and iii) parents provided written consent for their participation. All children in this community develop otitis media by 12 weeks of age and around 50% experience a perforated TM in the first year of life [4]. Children in this study were similarly prone to severe infections and all had been treated for otitis media in the past. Staff at the community health centre and otitis media researchers working on other projects made the initial diagnosis. Health centre staff examined children's ears when they presented to the children's clinic either unwell or for follow-up of a medical condition. Researchers examined children's ears as part of a regular 4 weekly surveillance program. Children with otorrhoea that had persisted for longer than six weeks were not eligible unless they had a diagnosis of AOM in the other ear. We attempted to assess children every weekday they were in the study. The planned duration of follow-up was either two weeks (AOM without perforation), or three weeks (AOM with perforation). We followed children with persistent AOM until the AOM resolved, or the study period ended.

### Clinical assessments

We used a questionnaire and review of the clinic notes to collect information about each child's current and past ear health. Wax and pus were removed from the ear canal under direct vision using a voroscope (WelchAllyn LumiView). A Siegel's speculum was used for pneumatic otoscopy. Images of the TM were video recorded and classified on a standardised data collection form. The position of the TM was described as retracted (R), neutral (N), mild bulging (B1), moderate bulging (B2), and marked bulging (B3). TMs that had a perforation that was associated with mild, moderate, or marked bulging (P1-P3) were considered to be functionally intact. Examples of these different positions and "pinhole perforations" are available in a training video [8]. Tympanometry (Grason Stadler GSI-38, Madison, Wisconsin, USA) was used in ears without any discharge. Tympanograms were classified according to a modified Jerger's classification [9]. Tympanograms with a canal volume of <0.3 cm^3 ^were excluded.

We classified children according to the ear with the more severe acute infection. When an assessment was only successful in one ear, we used the diagnosis of that ear and assumed the disease was unilateral. Generally, the person who made the initial diagnosis started antibiotic treatment before referring the child to this study. Medical or nursing research staff prescribed additional antibiotics during the study according to population-specific, evidence-based guidelines [10]. We calculated compliance with prescribed antibiotics on a weekly basis (the number of doses reported as taken divided by the number of doses prescribed for that week). All families were reminded about their medication at each assessment and some had help in dispensing medicine from Aboriginal research assistants.

### Definitions

Our classification of otitis media was based on current population-specific, evidence-based guidelines [10]. In this study (where close follow-up was ensured), we used the following criteria: i) Aerated middle ear-normal TM mobility and Type A or C tympanogram; ii) Otitis media with effusion (OME)-middle ear effusion behind an intact TM identified by an air-fluid level or bubble seen through a translucent TM or decreased mobility of the TM or type B tympanogram (admittance <0.2 mmho); iii) AOM without perforation-clinical diagnosis by health staff or moderate to marked bulging of an intact TM plus decreased TM mobility or Type B tympanogram; iv) AOM with perforation-evidence of recent TM perforation provided by clinical history and visualisation of fresh pus in the ear canal. The TM was intact if the perforation had already healed by the time of the assessment, and 'functionally intact' if the TM was bulging and the perforation could only be identified by pneumatic otoscopy; v) Chronic suppurative otitis media (CSOM)-TM perforation with otorrhoea present for more than six weeks; vi) Dry perforation-TM perforation with no discharge seen in the ear canal or within the middle ear space.

AOM was cured when there was no bulging and no discharge present on examination. Similarly, we described an episode of AOM as improved if: i) the ear discharge had resolved but bulging of the TM persisted in AOM with perforation, or ii) the bulging of the TM was reduced to mild in AOM without perforation. Examples of mild, moderate, and marked bulging of the TM are available in our training video [8]. The outcome at "1 week" was determined by the examination that was closest to day 7 (at least 5 days after the diagnosis). The outcome at "2 weeks" was determined by the examination that was closest to day 14 (at least 10 days after diagnosis).

### Microbiology: specimen collection and processing

We took swabs of the nasopharynx (and ear discharge if present) on up to five occasions: day 0 (the day of enrolment) and days 4–7, 10–14, 17–21 and 24–28. All swabs were then smeared for gram staining and frozen in 1.0 ml skim-milk-glucose-glycerol broth (SMGGB). We processed swabs (after completion of clinical observations) using standard methods that have been previously published [4]. We tested isolates of pneumococcus for sensitivity to oxacillin, penicillin, erythromycin, sulphamethoxazole, tetracycline, and chloramphenicol using a disc diffusion method (calibrated dichotomous susceptibility, CDS) [11]. Colonies resistant to oxacillin or penicillin were classified as penicillin resistant. Colonies resistant to three or more classes of antibiotics were classified as multi-resistant. Penicillin minimum inhibitory concentration (MIC) was determined by E-test and categorised according to the following breakpoints: susceptible <0.064 μg; intermediate resistance 0.064 – 1.0 μg/ml; and high resistance >1.0 μg/ml.

## Results

### Features of participants and examinations

We enrolled 31 children in this study and completed 219 assessments. Clinic staff referred 13 children and other researchers referred 18 children. Children referred from the clinic were more likely to have otorrhea (8/13 versus 6/18). The mean duration of follow up was 21 days (range 3–57 days). Most children were less than 2 years of age and had a past history of otorrhea (see Table 1). Overall, 230 tympanograms were recorded. Nearly all of these were Type B (223/230, 97%). Tympanograms were not done if ear discharge was present.

**Table 1:** Characteristics of study participants.

Characteristic	No. (%) of children
Number of children	31 (100%)
Male	15 (48%)
Age < 2 years	22 (71%)
Previous history of otorrhoea	22 (71%)
	
Otoscopy at start of study (per child):	
Redness	24 (78%)
Bulging (intact, no discharge)	15 (49%)
Otorrhoea (<6 weeks)	14 (45%)
Perforation seen (<6 weeks)	12 (39%)
Bilateral AOM	13 (42%)

### Initial diagnosis of AOM

Seventeen children had an initial diagnosis of AOM without perforation and 14 children had AOM with perforation. Nearly all children (27/31, 87%) had at least moderate bulging of the TM or recent discharge when they were first seen in this study; 13 had bilateral disease (42%). Symptoms of AOM could be assessed in 24/31 (77%) of study participants. Mothers reported otorrhea in 11/24 (46%) and ear pain in 7/24 (29%). Of the 14 children with AOM with perforation, two had a TM that appeared to have healed by the time of our examination and four had a TM that was functionally intact. All perforations seen were initially tiny in size (less than 2% of the area of the TM) and just antero-inferior to the centre of the TM.

### Antibiotic treatment

Six children were known to be receiving antibiotics effective against respiratory pathogens at the time of the initial AOM diagnosis (amoxicillin 50 mg/kg/day). All the other children had received antibiotics for otitis media in the past but the time since their last treatment was not recorded. Following diagnosis, antibiotic treatment was started in an additional 22 children. Two of the three children not treated initially developed moderate bulging of the TM and started treatment on day 6–8 (A31 and A32). One child (A19) did not receive any antibiotics. The antibiotics prescribed were twice daily amoxicillin (25), amoxicillin-clavulanate (1), trimethoprim-sulphamethoxazole (3), or daily intramuscular procaine penicillin (1). The mean dose of penicillin or amoxicillin was 52 mg/kg/day (standard deviation 15, range 25–100). Recommended treatment duration was at least 7 days for AOM without perforation and at least 14 days for AOM with perforation. Compliance with prescribed treatment was poor. Overall, we documented 462/1151 prescribed doses (40%) as taken, and estimated that 17/30 participants (57%) took less than half their recommended treatment.

### Clinical outcomes

AOM was persistent in most children from this high-risk population (see Table 2 and Table 3). Overall, 77% of children still signs of ongoing inflammation at the 7 day and 14 day assessments. Very few ears returned to normal. Of the 438 ear examinations attempted during this study, only 5 (1%) were consistent with an aerated, intact TM (A03 on 1 occasion, A19 on 1 occasion, and A29 on 3 occasions). Even when the acute infection resolved, recurrence was common. Children who received more than 50% of the prescribed antibiotics had similar rates of treatment failure to the children who did not (75% vs 71%, Risk difference 4%, 95%CI -33, 41). Similarly, children with a past history of otorrhea had similar rates of treatment failure to the children who did not (68% vs 75%, Risk difference -7%, 95%CI -43, 30). There were also no obvious differences in the clinical course of the six children who were already on antibiotics when they were enrolled in the study.

**Table 2:** Clinical outcomes at 7 days after diagnosis of acute otitis media (AOM). AOM was considered cured when there was no bulging and no discharge present on examination. An episode of AOM was considered improved if: i) the ear discharge had resolved but bulging of the TM persisted in AOM with perforation; or ii) the bulging of the TM was reduced to mild in AOM without perforation. Despite improvement, these children still had persistent signs of suppurative infection.

	Cure (%)	Improved (%)	Not Improved (%)
AOM without Perforation (n = 16)	6 (38%)	1 (6%)	9 (56%)
AOM with Perforation (n = 14)	1 (7%)	3 (21%)	10 (71%)

**TOTAL (n = 30)**	7 (23%)	4 (13%)	19 (63%)

**Table 3:** Clinical outcomes at 14 days after diagnosis of acute otitis media (AOM). See Table 2 for explanation of diagnostic categories.

	Cure (%)	Improved (%)	Not Improved (%)
AOM without Perforation (n = 14)	3 (21%)	1 (7%)	10 (71%)
AOM with Perforation (n = 12)	3 (25%)	1 (8%)	8 (67%)

**TOTAL (n = 26)**	6 (23%)	2 (8%)	18 (69%)

Seventeen children (55%) had a TM perforation documented at some point during the study. Of the 4 children with AOM with perforation who were followed for 6 weeks, 3 had healing and recurrence of their TM perforation documented. None of these four children had the typical otoscopic features of CSOM. In all cases the perforation was too small (usually a "pinhole") to allow adequate delivery of topical antibiotics to the middle ear space.

### Microbiological outcomes

Overall, 93 nasopharyngeal swabs (31 children) and 38 swabs of ear discharge (13 children) were taken during this study. Each child was eligible for a maximum of five swabs (mean 3). Most of the nasopharyngeal swabs were positive for *S. pneumoniae *(76/93, 82%), *H. influenzae *(66/93, 71%) or *M. catarrhalis *(86/93, 95%). All but two of the nasopharyngeal swabs were positive for at least 1 pathogen (98%), and 59/93 (63%) were positive for all three otitis media pathogens. Antibiotic sensitivity testing was done in 74 of the 76 nasopharyngeal swabs positive for *S. pneumoniae*. Of these, 59/74 (80%) were penicillin resistant and 23/74 (31%) were multi-resistant. E-tests were available for 44 of the 59 penicillin resistant isolates: 3/44 (7%) were highly penicillin resistant.

We were able to take an initial swab in 27/31 children. All but one of these swabs were positive for at least one pathogen, and 18/27 (67%) were positive for all three otitis media pathogens-see Table 4. The prescription of antibiotics did not reduce carriage of these pathogens at follow up assessments. Nasopharyngeal carriage of penicillin resistant pneumococci was common at diagnosis (16/27, 59%). This did not change substantially following treatment-see Figure 3. The carriage of penicillin resistant pneumococci was not associated with an increased rate of clinical failure (62% vs 80%, Risk difference -18%, 95%CI -62, 25).

**Table 4:** The proportion of children with bacterial pathogens isolated from their nasopharynx.

	Spn (%)	PRSpn (%)	Hi (%)	Mc (%)
**Day 0**	22/27 (81%)	16/27 (59%)	19/27 (70%)	26/27 (96%)
**Week 1**	20/24 (83%)	16/24 (67%)	17/24 (71%)	24/24 (100%)
**Week 2**	18/21 (86%)	13/21 (62%)	17/21 (81%)	20/21 (95%)

Spn = *Streptococcus pneumoniae*; PRSpn = Penicillin Resistant *Streptococcus pneumoniae*; Hi = *Haemophilus influenzae*; Mc = *Moraxella catarrhalis*.

Identification of nasopharyngeal pathogens from swabs of ear discharge was less common: *S. pneumoniae *11/38 (29%), *H. influenzae *12/38 (32%), or *M. catarrhalis *2/38 (5%). Nineteen ear discharge swabs (50%) were positive for at least one of these pathogens and 1/38 (3%) was positive for all three otitis media pathogens.

### Onset and progression of AOM with perforation

**Figure 1:**
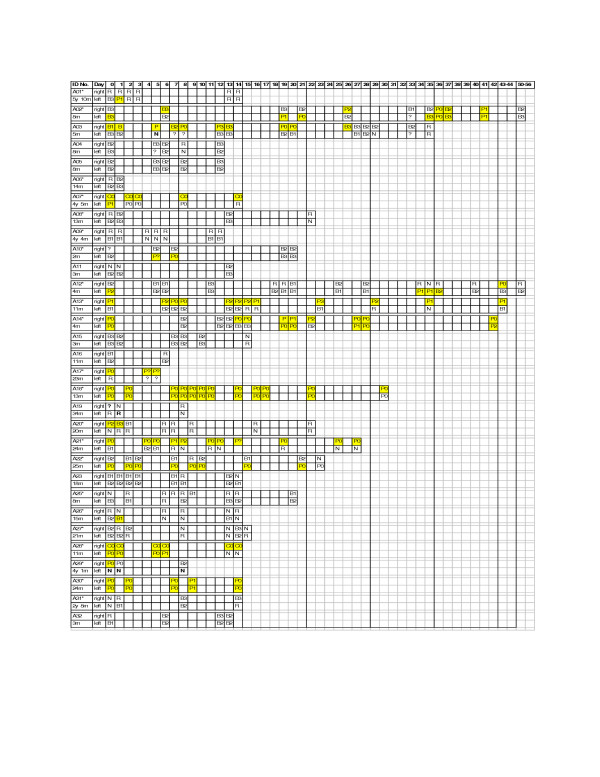
**Position and integrity of tympanic membrane in 31 Australian Aboriginal children following a new diagnosis of acute otitis media. **Shaded = discharge present; C = chronic perforation; P = acute perforation; B = bulging; n = neutral; R = retracted; 0 = no bulging; 1 = mild bulging; 2 = moderate bulging; 3 = marked bulging. * = past history of otorrhea.

The changes in TM position and diagnosis over the 219 examinations are shown in Figure 1. Of the 14 children who entered the study with a diagnosis of AOM with perforation, two TMs had already healed and six were functionally intact (ie. pinhole perforation only seen on pneumatic otoscopy). Both children who initially had fresh discharge obscuring an intact TM subsequently experienced recurrent perforations (A02 and A03). In the other 12 children with identifiable perforations, discharge continued seeping through the perforation almost immediately after completing the cleaning or swabbing of the middle ear canal. Eight TMs were initially perforated and in the neutral position. Over time, five of these also became functionally intact (ie. the perforation size reduced and the TM became bulging). AOM without perforation progressed to perforation in three children (A01, A10 and A26). In each case, the TM was observed to be bulging prior to perforation. On one occasion when a new perforation was observed, the TM had perforated and healed again within a 24 hour period (A26).

There were two patterns of resolution of AOM with perforation. In four children, the suppurative process resolved and the perforation became dry (A07, A18, A22, A29). Two of these TMs subsequently healed during the study period. More commonly, the perforation appeared to be healing while the suppurative process was ongoing (A01, A02, A03, A10, A12, A14, A20, A26, and A28). Perforations were observed to heal and re-perforate in 4 children (A02, A03, A12, and A14). In one child, the right and left TM healed and re-perforated 8 times over a 6 week period (A02), confirming that healing and re-perforation can occur frequently.

## Discussion

This was the first study describing the clinical course of AOM in Australian Aboriginal children. It is also the first study providing a detailed description of otoscopy findings in a population of high-risk of TM perforation. AOM (or suppurative OM) was common, usually not associated with ear pain, frequently bilateral, and often associated with perforation of the TM. In this population, AOM was generally persistent. Infections with a sudden onset and short duration were uncommon.

### Strengths and limitations of the study

We used a standardised clinical assessment that included tympanometry and video-otoscopy. Since definitions of AOM vary [12,13],. the detailed description of the position and integrity of the TM over time is especially useful (see Figure 1). This information has not been reported previously.

Limitations of the study include the small number of children enrolled and the number of scheduled examinations that were missed. This is a consequence of performing research in remote Aboriginal communities. It reflects the challenges associated with research in remote locations with small populations. Participating families are culturally different, highly mobile, and have competing priorities that are not easily predicted. These factors make daily follow-up difficult. The small sample size and relative homogeneity of the study population mean that potentially important factors that predict outcome may no be identified. However, a larger study (or observation over a longer duration) is unlikely to change our conclusion that "persistent AOM" is common in this population (overall risk of persistent AOM 77% 95%CI 58, 90). Since there are currently no data available in the world on the clinical course of AOM in a population at high-risk of AOM, the information contained within Figure 1 is both unique and clinically important.

The high rates of nasopharyngeal carriage of all three respiratory pathogens are striking. The microbiological methods used in this small study cannot determine i) which pathogens extend from the nasopharynx to the middle ear space; ii) the relative importance of concurrent infection with multiple organisms; iii) the rate of acquisition of new pathogens while on treatment; or iv) the role of antibiotic resistant bacteria. While we did not find pneumococcal penicillin resistance of organisms in the nasopahrynx to be a good predictor of outcome, the small sample size mean that important effects cannot be excluded.

### Comparison with outcomes in clinical trials

Should we be surprised that "persistent AOM" is so common in this population? In published prospective studies where children have not been treated with antibiotics, the rate of clinical failure in the first week ranged from 2% to 83% [14-23]. The median failure rate was 24%. The most striking influence on reported rates of clinical failure was the definition used for this outcome. Authors that defined clinical failure in terms of persistent symptoms (fever or otalgia) reported the lowest rates of clinical failure (all <25% at the end of the first week) [14,16-19]. The median clinical failure rate for these studies was 15%. In contrast, the five studies with the highest clinical failure rates (range 38–83%, median 73%) defined clinical failure as persistence of otoscopic signs [15,20-23]. Only one study described both the rate of persistent symptoms (13%) and persistent otoscopic signs (73%) after seven days [22]. Overall, these studies suggest that symptoms resolve quickly in most children with AOM not receiving antibiotics while otoscopic signs do not. Consequently, we should probably not be surprised by our findings of persistent otoscopic signs in a high-risk population where compliance with recommended antibiotic treatment is poor and antibiotic resistance is common.

Unfortunately, in nearly all published clinical trials, there is insufficient information to determine whether persistent bulging of the TM was a common finding [24]. Persistent middle ear discharge was probably unusual (as it is a readily identified complication of AOM). None of the studies described the outcomes specifically for the subgroup of children who had AOM with perforation at the time of diagnosis. Similarly, none of these studies described the associated nasopharyngeal colonisation during episodes of AOM. However, other studies in developed countries have found that dense colonisation with multiple bacterial pathogens is unusual [25]. This may be an important risk factor for the children included in this study.

### Implications of the study

For populations where perforation of the TM and CSOM are uncommon, this study provides important information about: i) appropriate definitions for different types of OM; and ii) identification of individual children most at risk of CSOM. For high-risk populations, we believe our description of "persistent AOM" is likely to be generalisable. This persistence may be related to the severity of clinical presentation, the bacterial load (multiplicity of species and strains and their density of infection), the frequency of exposure to multiple pathogenic strains, or poor compliance with antibiotics. The early age of onset of suppurative ear infections and the low rates of reported symptoms make early recognition difficult. Consequently, active surveillance in all infants in this population is recommended.

## Competing interests

The author(s) declare that they have no competing interests.

## Authors' contributions

All the authors contributed to the development of the study protocol and the writing of the paper. As part of her BMedSci, KG initiated the study protocol, collected and analysed the data, and wrote the first draft of the manuscript. PM, JC, SS, and AL supervised KG and assisted with the design and analysis of the study. PM, JC, SS, and AL were responsible for early revisions of the manuscript. PM, AL, and ES assisted with data collection. AL, ES, and HSV supervised the microbiological aspects of the study that were completed by their technical staff. All authors read and approved the final manuscript.

## Pre-publication history

The pre-publication history for this paper can be accessed here:

http://www.biomedcentral.com/1471-2431/5/16/prepub
